# Dr. Pettey’s Western Retreat

**Published:** 1904-09

**Authors:** 


### DR. PETTEY'S WESTERN RETREAT.

Having met with signal success in his Memphis Institution for the treatment of alcohol and drug addictions, and since there is a strong demand for institutions of this kind conducted within ethical lines, Dr. Pettey has opened a Retreat at 1939 East Evans Avenue, Denver, Colo., and will soon open another at Atlantic City, N. J.

These institutions will be under the management of physicians who have been associated with Dr. Pettey in this line of work in his Memphis Retreat and who are thoroughly familiar with his methods of treatment and with the care and management of this class of cases. It has now been three years since Dr. Pettey published to the profession a successful treatment for the Narcotic Drug Addictions and, he says, since others seem so slow to prepare for and take up this line of work, he feels justified in attempting to meet the demand for treatment by the methods he has introduced by opening these additional institutions. He assures the profession that they can depend upon the treatment at his Branch Retreats being kept up to the same standard as that administered under his immediate supervision.

Dr. Pattey is one of the few physicians engaged in this line of work who does not advertise to the public direct and whose methods are entirely open and ethical. We bespeak for his Eastern and Western Retreats the same hearty professional support that has been given his Memphis Retreat.

